# Nutrition for Podocyte Repair in Nephrotic Syndrome?

**DOI:** 10.3390/nu15214615

**Published:** 2023-10-31

**Authors:** Roberto Aquilani, Manuela Verri

**Affiliations:** Department of Biology and Biotechnology “Lazzaro Spallanzani”, University of Pavia, 27100 Pavia, Italy; dottore.aquilani@gmail.com

Nephrotic syndrome (NS) poses a number of nutritional and metabolic problems due to glomerulus injured podocytes, which are responsible for the loss of barrier function, causing proteinuria, altered fluid and electrolyte balances, and hypoalbuminemia. In addition, these patients may present with macro-micronutrient deficits, hyperlipidaemia, growth retardation (in paediatric patients), and malnutrition.

So far, nutritional interventions have been adopted to improve these metabolic alterations, in particular, in paediatric patients [1]. However, nutrition could also be potentially adopted to limit podocyte stress and damage by acting mainly on podocyte mitochondrial dysfunction [2], oxidative stress [2,3,4,5], and protein dysmetabolism [6].

Some crucial considerations for this hypothesis are as follows: first, podocytes are rich in mitochondria [2,7] and consume a lot of energy [2]. Altered mitochondrial populations, functions, and dynamics have been found in early diabetic nephropathy and other glomerular diseases, including focal segmental glomerulosclerosis (FSGS) [8,9,10,11]. Mitochondrial alterations may lead to an energy production deficit and increased reactive oxygen species (ROS) formation. Both impaired energy generation (ATP) and increased ROS could induce podocyte net protein catabolism due to reduced protein synthesis from low-level ATP and increased protein catabolism from ROS.

Second, ROS, in addition to damaging the mitochondria and cell membranes, activate proteolytic pathways [12,13]. The oxidative stress level is high, particularly in childhood NS [4].

Third, mitochondrial protein dysmetabolism may be due to the degradation of proteins that constitute the podocyte structures, such as prohibitin-2 [14], slit membrane components, glomerular basement membrane (GBM) components, and the anchor components between the cytoskeleton and the GBM [15].

Thus, net protein catabolism in podocytes could lead to a persistent podocyte injury [6] and may aggravate glomerular diseases.

With the aim of potentially limiting the above podocyte metabolic abnormalities, nutrition intervention should be conducted with caution, as excessive energy and protein intakes might be inefficient and detrimental to mitochondrial functionality (Table 1 and Table 2) [16,17].

The excess of protein intake deserves focused attention as the amount of amino acid-activated mTORC1 (mammalian target of rapamycin complex) is increased in glomerular podocytes in human and animal diabetic nephropathy [18,19]. In contrast, the inhibition of mTOR increases the population of damaged mitochondria by suppressing podocyte autophagy, leading to proteinuria [20].

Therefore, from a nutritional standpoint, reconciling these two opposite metabolic conditions is a problem. As a working hypothesis, substituting protein an equivalent amount of essential amino acids could reduce the amino acid burden on mTOR, hence reducing the hyperstimulation of mTOR, and could contribute to improving the rate of liver albumin synthesis [21,22]. Indeed, the essential amino acid tryptophan limits albumin synthesis [21,22]. In addition, essential amino acids could improve the syntheses of other proteins, such as transferrin, erythropoietin, and haemoglobin, that, in NS, are lost in urine.

Of interest, essential amino acids can induce mitochondriogenesis, mitochondrial activation, and reduced ROS formation [23,24,25].

This Special Issue aims to act both as a stimulus for future research regarding the possible influences that nutrition could exert on limiting/repairing podocyte damage and dysfunction and as a means to correct the main nutrition alterations in clinical practice.

## Figures and Tables

**Table 1:** Some potentially negative effects of excessive energy intake on dysfunctional mitochondria.

(1) Increase in ROS formation.
(2) ROS-driven destruction of mitochondria.
(3) ROS-driven activation of the inflammatory pathway.
(4) Attenuation of autophagy and mitophagy.
(5) Excess of dietary carbohydrates and lipids may be inefficiently used by mitochondria metabolic pathways.

**Table 2:** Some potentially negative effects of excessive protein intakes on podocyte metabolism.

Excess of Amino Acids Raises Ammonia Concentrations That Are Toxic to:
(1) Mitochondrial function.
(2) Protein synthesis.
(3) Muscle function and structure → increased muscle autophagy (sarcopenia).
